# The association between Chorionic Villus Sampling and preterm birth: A systematic review

**DOI:** 10.1093/eurpub/ckac131.421

**Published:** 2022-10-25

**Authors:** K Giannakou, L Mastrou

**Affiliations:** Department of Health Sciences, European University Cyprus, Nicosia, Cyprus

## Abstract

**Background:**

Preterm birth is defined as a syndrome that can be initiated via various mechanisms. During the past few decades, it has been an emerging health issue worldwide and thus, several actions to benefit public health have been undertaken to reduce its rate. Chorionic Villus Sampling (CVS) is one of the methods that are performed prognostically for prenatal diagnosis. However, several complications can occur after this invasive method is performed. The aim of this review is to identify whether CVS may result in preterm birth as a complication, compared to other invasive or non-invasive methods.

**Methods:**

A systematic literature review was conducted in PubMed and Scopus from inception until December 2021, to identify studies examining the above association. The research strategy included a combination of search and MESH terms related to CVS and preterm birth, Inclusion and exclusion criteria were set. The research was conducted by two researchers that thoroughly screened the articles and extracted the data.

**Results:**

37 studies met the inclusion criteria. Comparisons were performed between CVS and amniocentesis or control group or between the different weeks that CVS was performed. No significant difference was observed in most of the studies when CVS was compared to amniocentesis, control groups, or between different weeks of gestation that was performed.

**Conclusions:**

The risk of preterm birth of women undergoing CVS was relatively lower or had no difference compared to the other methods or controls investigated. Therefore, CVS is considered as a safe prenatal diagnostic procedure with minimum rates of preterm birth as a complication.

**Key messages:**

• The risk of preterm birth of women undergoing CVS was found to be relatively lower with no significant differences compared to the other methods or controls.

• Future studies should be performed recruiting a large sample with women of different age and compare it with a control group that will not undergo any invasive method.

